# Impact of Short-Term Systemic Hypoxia on Phagocytosis, Cytokine Production, and Transcription Factor Activation in Peripheral Blood Cells

**DOI:** 10.1155/2011/429501

**Published:** 2011-06-02

**Authors:** Michael Fritzenwanger, Christian Jung, Bjoern Goebel, Alexander Lauten, Hans R. Figulla

**Affiliations:** Department of Internal Medicine I, Division of Cardiology, Friedrich-Schiller-University, Erlanger Allee 101, 07740 Jena, Germany

## Abstract

Hypoxia frequently associated with certain physiologic and pathologic conditions influences numerous cellular functions. Because the effects of short-term hypoxia are incompletely understood, we examined phagocytosis and cytokine production as well as the activation of the transcription factors HIF-1 and NF*κ*B in peripheral blood cells of healthy volunteers exposed to an oxygen concentration equivalent to that found at a height of 5500 m. Furthermore, we analysed plasma HIF-1 and serum concentrations of various HIF-1-dependent genes. Results showed that short-term hypoxia increased phagocytosis in neutrophils without affecting monocyte phagocytosis. Hypoxia decreased basal TNF*α* concentration in monocytes and basal interferon *γ* concentration in CD4^+^ T lymphocytes. In contrast, plasma HIF and serum VEGF concentrations were not affected by hypoxia, although serum EPO concentration was raised. In PBMC, hypoxia increased cytosolic HIF-1 concentration without affecting nuclear HIF-1 concentration and led to a rise in the nuclear NF*κ*B in PBMC. Our results show that short-term hypoxia affects immune functions in healthy individuals. Furthermore, we speculate that the effects of hypoxia are not due to HIF-1, but are caused by the activation of NF*κ*B .

## 1. Introduction

Several physiological and pathological states can cause systemic or localised tissue hypoxia and accordingly all nucleated cells can sense and respond to changes in the oxygen concentration [1]. The organism's response to hypoxia is multifactorial involving the haematopoietic, respiratory, and cardiovascular systems in order to maintain adequate tissue oxygenation. However, severe hypoxia causes oxidative stress in blood [2, 3]. Additionally, hypoxia induces a microvascular inflammatory response leading to increased vascular permeability and leukocyte-endothelial adherence and emigration [4, 5]. 

Phagocytosis is important for the organism in the defence against invading microorganisms and in ridding the body of dead cells [6]. Phagocytes include several cell types such as neutrophils, monocytes, macrophages, dendritic cells, and mast cells. A disturbed phagocytosis, found in several diseases, results in increased susceptibility to bacterial infections [7, 8]. A study by Wang and Liu carried out on healthy volunteers exposed to a 12% oxygen concentration demonstrated that hypoxia increased chemotaxis, phagocytosis and respiratory burst in leukocytes [9]. The study group also showed that moderate exercise performed under an oxygen concentration of 12% enhanced phagocytosis and promoted apoptosis of neutrophils [10].

At the cellular level, the majority of genes expressed after hypoxia are regulated by the hypoxia-inducible factor-1 (HIF-1), a heterodimer consisting of an *α* and *β* subunit. Under normoxic conditions, HIF-1*α* is rapidly degraded by proteasomes. However, under hypoxic conditions, HIF-1*α* does not undergo degradation; instead, it functions as a transcription factor for various genes involved in angiogenesis, vasomotor control, maturation of red blood cells, energy metabolism, cell proliferation, and viability [11]. 

 Erythropoietin (EPO), a glycoprotein that is mainly produced in the proximal tubular cells of the kidney and to a small extent in the liver, is one of the genes regulated by HIF-1 [12, 13]. Whereas, under physiological conditions, EPO regulates the manufacture of red blood cells, the production of EPO itself is induced by a hypoxic and reduced by hyperoxic state. 

Vascular endothelial growth factor (VEGF) constitutes another HIF-1-regulated gene. VEGF, a homodimeric, heparin-binding glycoprotein, has angiogenic, mitogenic, and vascular permeability-enhancing properties especially for endothelial cells. VEGF expression is found in activated macrophages [14], keratinocytes [15], renal glomerular visceral epithelium, and mesangial cells [16, 17] as well as other cells types including many tumor cells.

In contrast to the extensive knowledge regarding phagocytosis of neutrophils under hypoxia, monocyte phagocytosis as well as cytokine expression of monocytes and T lymphocytes under hypoxic conditions is not well understood. For example, mouse T lymphocytes under hypoxia showed an increased expression of interleukin-2, -4 and interferon *γ* in cell culture [18]. Dziurla et al. [19] also found an increased expression of interleukin-2, -6, -8, -10, and -1*β*, and the monocyte chemoattractant protein-1 in PMA/ionomycin stimulated human monocytes exposed to hypoxia *in vitro*. 

In the present study carried out on healthy volunteers exposed to hypoxia in a specialised hypoxia chamber, we examined for the first time: (1) phagocytosis of neutrophils and monocytes; (2) cytokine expression of monocytes and CD4^+^ T lymphocytes; (3) serum concentrations of HIF-1, EPO and VEGF; (4) the activation of HIF-1 and NF*κ*B in peripheral blood mononuclear cells.

## 2. Methods

### 2.1. Subjects

The study was reviewed and approved by the Ethics Committee of the Friedrich-Schiller-University, Jena, Germany. All volunteers gave their written informed consent. All experiments were performed according to institutional guidelines. Subjects were recruited exclusively from the staff of the Department of Internal Medicine I of the Friedrich-Schiller-University, Jena, Germany. Fourteen obviously healthy volunteers enrolled in this study (2 females and 12 males). The characteristics at baseline and at an oxygen tension equivalent to an altitude of 5500 m are shown in Table 1. Serum parameters between normoxia and hypoxia were not different despite a small tendency towards an increased lactate concentration under hypoxic conditions (Table 2). We did not find a significant difference between the ratio of monocytes/lymphocytes/neutrophils under normoxic or hypoxic conditions (data not shown).

### 2.2. Study Protocol

**Figure 1:**
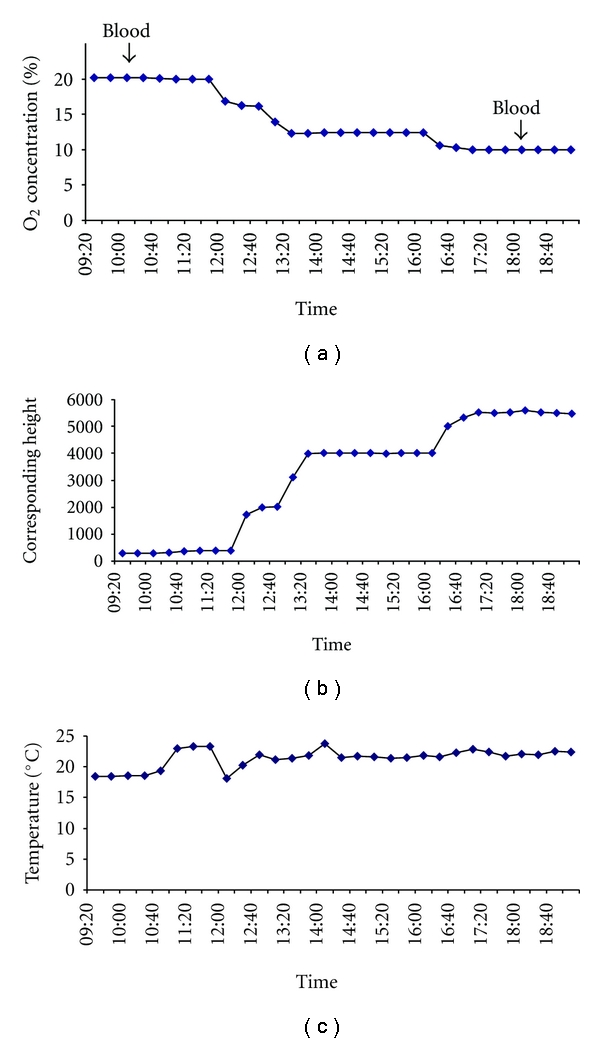
Time course of oxygen concentration and corresponding height and temperature in the hypoxia chamber during the experimental period. Times of blood collections are indicated by arrows.

The study was performed in an air-conditioned normobaric hypoxia chamber which contained a carbon dioxide (CO_2_) scrubber to eliminate CO_2_. The time-dependent oxygen and concentration corresponding height and temperature in the chamber are shown in Figure 1. The first blood sample was obtained following stabilization. After 2 hours, the oxygen concentration of the chamber was adjusted to a value equivalent to a height of 4000 m. This value was maintained for 4 hours to allow for adaption. The oxygen concentration was then adjusted to a value equivalent to a height of 5500 m to achieve hypoxic conditions with a peripheral O_2_ saturation of around 75%. A second blood sample was taken after two hours of acclimatisation.

### 2.3. Blood Samples

Blood samples were collected from an antecubital vein using a clean venipuncture under controlled venous stasis. The first 2 mL of blood was discharged, and the remaining blood was used to measure hematological parameters and blood cell functions. For safety reasons, the test was terminated immediately when a subject's level of O_2_ saturation dropped to <70%, or when the subject complained of discomfort. All subjects were free of symptoms of acute mountain sickness during the experimental period. A 100% compliance rate was obtained for the study.

### 2.4. O_2_, Heart Rate, and Blood Lactate Measurements

Peripheral O_2_ saturation (SaO_2_) was measured by means of finger pulse oximetry (Masimo Radical-7, Masimo Corp., Calif, USA); blood pressure (BP) and heart rate (HR) were monitored using an automatic blood pressure system. Finally, capillary blood lactate was measured employing a common blood gas analyzer (Ciba Corning 865, Chiron Diagnostics, Norwood, Mass, USA).

### 2.5. Serum Electrolyte, Glucose, and Lactate Determination

Serum electrolyte, glucose, and lactate concentrations were determined using an ABL 800 (Radiometer, Germany).

### 2.6. Plasma HIF-1, Serum EPO, and VEGF Concentration

Plasma HIF-1, serum EPO, and VEGF serum concentrations were determined by commercial available ELISA (HIF-1 CUSABO113 Barksdale Professional Center Newark, DE19711, EPO and VEGF RandD Systems, Germany) according to the manufacturers' instructions.

### 2.7. Phagocytotic Activity of Neutrophils and Monocytes

*FITC-labeling of zymosan*. 10 mg zymosan (Sigma Chemicals, Germany) were suspended by repeated sonification in 1 mL distilled H_2_O. 1 mg FITC (Sigma Chemicals, Germany) together with a small amount of NHCO_3_ (Sigma Chemicals, Germany) was dissolved in the zymosan solution and incubated at 40°C for 30 min. Thereafter, the FITC-labeled zymosan was washed three times with distilled H_2_O. Zymosan particles were counted and a working solution of 125.000 particles/*μ*L was prepared in phosphate-buffered saline. To determine phagocytic activity of neutrophils and monocytes, 500 *μ*L whole blood was mixed with 500 *μ*L FITC-labeled zymosan with or without phorbol 12-myristate 13-acetate (PMA, end concentration 10^−9^ M, Sigma Chemicals, Germany). After 20 and 60 min, 200 *μ*L of this solution were removed and erythrocytes were lysed with 2 mL ice-cold NH_4_Cl for 5 min. Subsequently, FITC positively stained neutrophils and monocytes were determined by flow cytometry. Measuring unspecific binding of FITC-labeled zymosan was abolished by quenching with trypan blue.

### 2.8. Immunofluorescent Flow Cytometric Analysis of Cytokine Production

For intracellular staining, peripheral blood was collected in lithium-heparin tubes. 100 *μ*L blood was immediately added to RPMI-1640 medium including brefeldin A (final concentration: 1 *μ*g/mL) and incubated for 6 hours at 37°C under 21% oxygen with or without PMA (concentration 10^−9^ M)/ionomycin (concentration 10^−7^ M) (PMA/iono). All chemicals were obtained from Sigma Chemicals, Germany. Next, erythrocytes were lysed by NH_4_Cl. After washing with PBS/2% FCS, cells were stained with monoclonal antibodies against the surface antigens CD3 (Coulter-Immunotech, Krefeld, Germany) and CD4 (Caltag, Hamburg, Germany) (15 min, RT), followed by a washing step and fixation with 100 *μ*L 2% paraformaldehyde (Sigma Chemicals, Germany) for 10 min at room temperature. After another wash, the cells were incubated in 100 *μ*L permeabilisation solution (0.1% saponin and 0.1% NaN_3_ in PBS) together with 1 *μ*L directly conjugated anticytokine antibodies (interleukin (IL)-1*β*, -2, -4, -5, -10, -17, interferon *γ* (IFN), and tumor necrosis factor (TNF)*α*, all from BD-Pharmingen, Heidelberg, Germany, for 15 min at room temperature. Followed by a second wash with permeabilisation solution, the cells were resuspended in PBS/2% FCS and fluorescence intensity was analysed by flow cytometry (FACSCalibur, Becton-Dickinson, Heidelberg, Germany). For analysis, regions were defined by forward scatter and side scatter. Data were analysed with CellQuest Software.

### 2.9. HIF-1*α*, p50, and p65 Activation in PBMC

Nuclear extracts were acquired by employing the EpiQuik Nuclear Extraction KIT I (Epigentek, NY, USA) according to the manufacturer's manual. Thereafter, protein concentrations of nuclear extracts were determined according to the Bradford method. For determination of HIF-1*α*, p50, and p65, 10 *μ*g of nuclear proteins were used. HIF-1*α*, p50, and p65, activation was determined by TransAM HIF-1 (Active Motif Europe, Belgium) or TransAM NF*κ*B family (Active Motif Europe, Belgium) ELISA according to the manufacturer's instructions. 

### 2.10. Statistical Analysis

All measurements are expressed as mean ± SD, with the exception of flow cytometric measurements which are expressed as mean ± SEM. Differences of a variable between hypoxia and normoxia were tested by the paired *t*-test. Bivariate regression analysis is shown in graphical form, and correlations were examined by Pearsons correlation test. For statistical analysis, WinSTAT and EXCEL were used. A value of *P* < .05 was accepted as significant.

## 3. Results

### 3.1. Hypoxia-Induced Phagocytosis of FITC-Labeled Zymosan in Neutrophils and Monocytes

**Figure 2:**
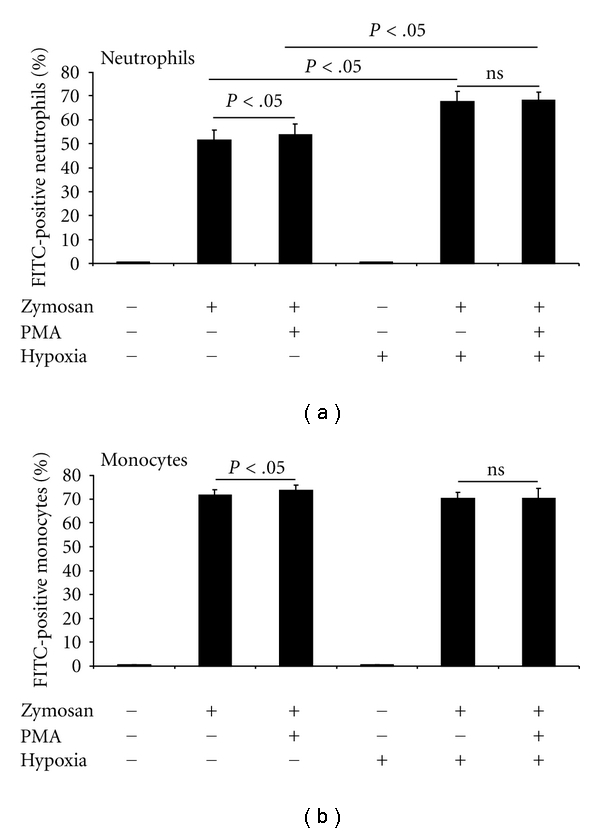
Phagocytic activity of neutrophils and monocytes. Neutrophils or monocytes under normoxia or hypoxia unstimulated or stimulated with PMA were examined. Data are shown as mean ± SEM; *n* = 14.

Phagocytosis in neutrophils and monocytes in whole blood was obtained before and after hypoxia equivalent to an altitude of 5500 m after 2 hours (Figure 2). The application of zymosan caused a dramatic increase of FITC positively stained neutrophils and monocytes independent of oxygen concentration. Under normoxia, PMA further increased the number of FITC-positive neutrophils and monocytes. Hypoxia caused a significant increase of phagocytosis in neutrophils. However, PMA did not influence this effect. In monocytes, hypoxia did not affect the uptake of zymosan independent of PMA. Moreover, we did not find a significant difference between phagocytosis measured after 20 min or after 60 min. These results demonstrate that *in vivo* hypoxia increased phagocytosis of neutrophils without influencing phagocytosis of monocytes.

### 3.2. Hypoxia-Induced Cytokine Production of Monocytes and CD4^+^ T lymphocytes

**Figure 3:**
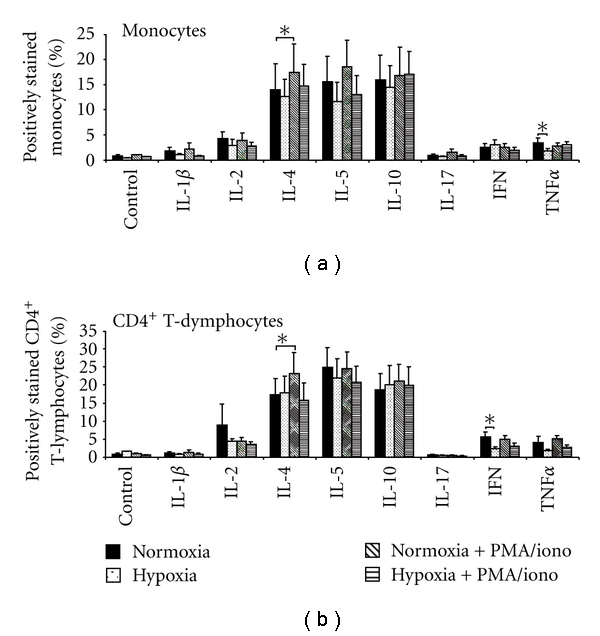
Effect of hypoxia and PMA/iono on the number of monocytes or CD4^+^ T lymphocytes stained positive for several cytokines determined by flow cytometry. The number of cytokine positive monocytes or CD4^+^ T lymphocytes was analysed by flow cytometry. *n* = 14; data are expressed as mean ± SEM. * *P* < .05.

In the next set of experiments, we tested whether hypoxia changed cytokine production of monocytes and CD4^+^ T lymphocytes. PBMCs obtained in normoxia and after stable hypoxia equivalent to a height of 5500 m for 2 hours were used (Figure 3). In unstimulated PBMC, hypoxia caused a decrease of TNF*α* in monocytes and a decrease of IFN in CD4^+^ T lymphocytes. We found no significant changes of the other tested cytokines either in monocytes or in CD4^+^ T lymphocytes after hypoxia. Stimulation with PMA/iono abolished the effect of hypoxia. Under normoxic conditions, PMA/iono significantly induced IL-4 both in monocytes and CD4^+^ T lymphocytes.

### 3.3. Plasma HIF-1, EPO, and VEGF Concentrations under Hypoxia

**Figure 4:**
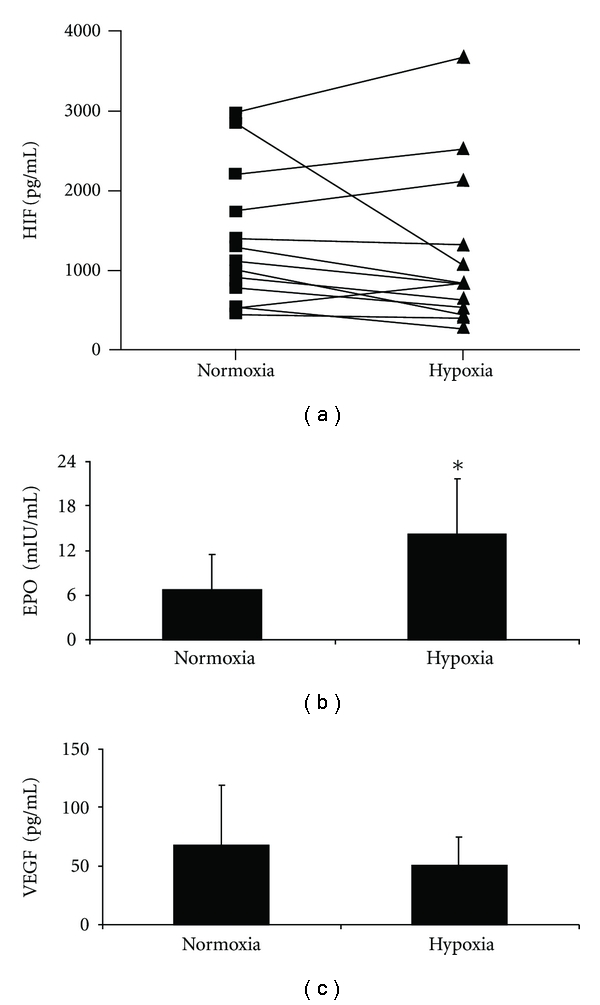
Plasma HIF, serum EPO, and VEGF concentrations under normoxia and hypoxia. *n* = 14, data are expressed as mean ± SD. * *P* < .05.

Figure 3 shows plasma HIF-1, EPO, and VEGF concentrations under normoxic and hypoxic conditions. HIF-1 plasma concentration was not influenced by hypoxia (Figure 4(a)) whereas EPO concentrations (Figure 4(b)) significantly increased during the experiment from 6.7 ± 4.7 mIU/mL to 14.2 ± 7.4 mIU/mL. Plasma VEGF concentrations (Figure 4(c)) were not influenced by short-term hypoxia. These data indicate that in healthy volunteers subjected to 6 hours of hypoxia in a hypoxia chamber, only the concentration of EPO and not that of VEGF increased, although both proteins are regulated by HIF-1. Plasma levels of HIF-1 were not affected during the experiment.

### 3.4. HIF-1*α* and NF*κ*B Activation in PBMC by Hypoxia

**Figure 5:**
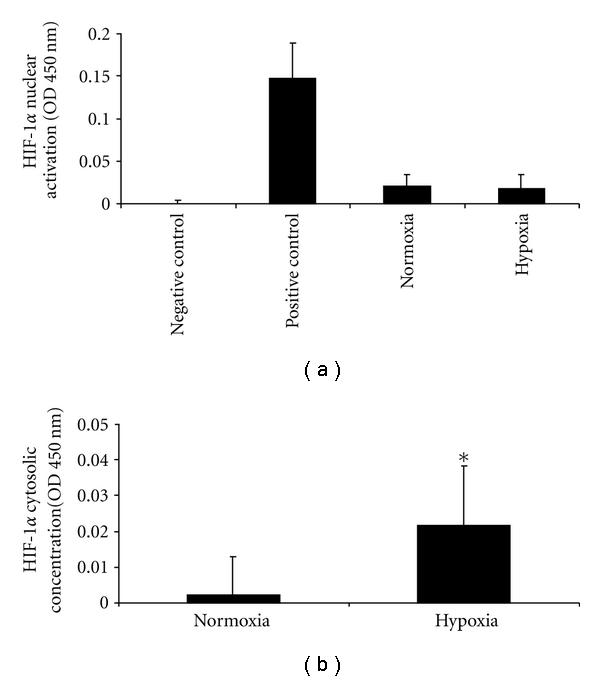
(a) Nuclear HIF-1*α* activity of PBMC under normoxia and after hypoxia. (b) Cytosolic HIF-1*α* concentration in PBMC. *n* = 14, data are expressed as mean ± SD.

**Figure 6:**
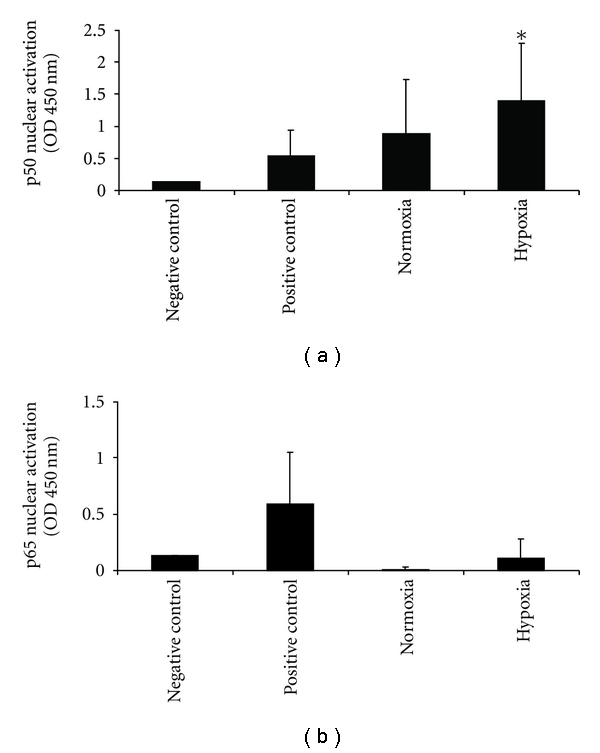
(a) Nuclear p50 activation in PBMC under normoxia and after hypoxia. (b) Nuclear p65 activation PBMC under normoxia and after hypoxia. *n* = 14; data are expressed as mean ± SD.

Because we found a significant reduction of TNF*α* in monocytes and INF in CD4^+^ T lymphocytes under hypoxia, we examined whether HIF-1*α* was activated due to hypoxia. Nuclear extracts of PBMC under normoxia and after hypoxia were prepared, and HIF-1*α* activity was determined with TransAM HIF-1. Results showed no HIF-1*α* translocation into the nucleus after hypoxia, although a significantly increased concentration of HIF-1*α* protein was detected in the cytosol following hypoxia. Our data indicate that under mild hypoxia, PBMC synthesis increased HIF-1*α* protein in the cytosol whereas translocation into the nucleus was not increased (Figure 5). Because hypoxia is also capable of activating the NF*κ*B pathway, we examined whether our experimental conditions caused activation of p50 or p65. For this purpose, nuclear extracts of normoxic or hypoxic PBMC were analyzed using the TransAM NF*κ*B family ELISA. In PBMC, hypoxia caused a significant nuclear translocation of p50 whereas p65 increased under hypoxia but did not reach significance (Figure 6) indicating that an oxygen concentration of 10% in the air caused an activation of the NF*κ*B pathway.

### 3.5. Serum Lactate Concentrations Correlate with Phagocytosis in Neutrophils and INF Expression in CD4^+^ T Lymphocytes

**Figure 7:**
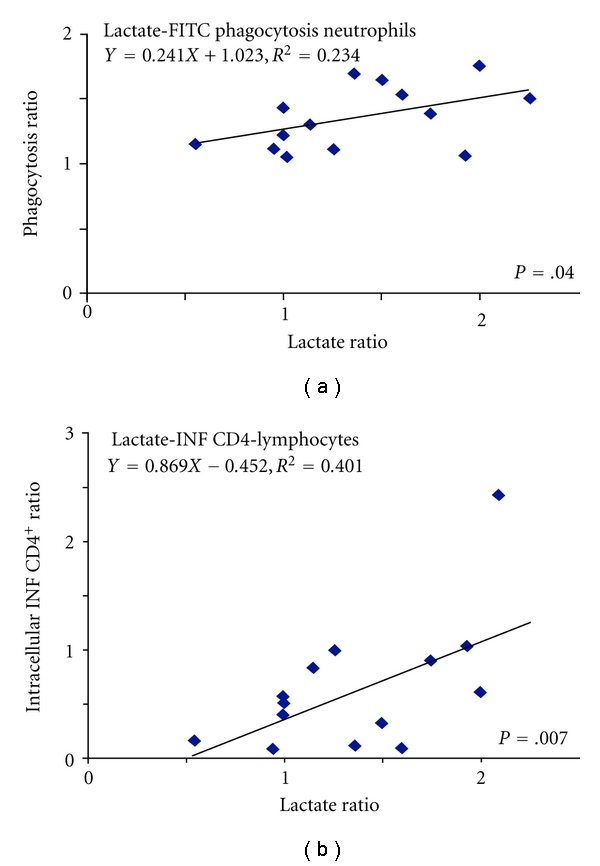
Correlation analysis determined a linear regression between (a) the lactate hypoxia/lactate normoxia ratio and the phagocytosis hypoxia/phagocytosis normoxia ratio in neutrophils, (b) the lactate hypoxia/lactate normoxia ratio and the intracellular INF hypoxia/intracellular INF normoxia ratio in CD4^+^ T lymphocytes.

We performed a correlation analysis and found a linear relation between the hypoxia/normoxia ratio of lactate concentrations and the hypoxia/normoxia ratio of phagocytosis of neutrophils measured by FITC-labeled zymosan incorporation (*Y* = 0.241*X* + 1.023, *R* ^2^ = 0.234, *P* = .040) (Figure 7(a)) and between the ratio of serum lactate concentration and the ratio of the expression of INF in CD4^+^ T lymphocytes (*Y* = 0.869*X* − 0.452, *R* ^2^ = 0.401, *P* = .007) (Figure 7(b)). This data indicates that despite serum lactate concentrations always being in a normal range, the lactate hypoxia/normoxia ratio correlated with neutrophil phagocytosis independent of PMA stimulation and INF expression in CD4^+^ T lymphocytes. We did not find any correlation between the other determined variables.

## 4. Discussion

The present study provides new insights into the effects of hypoxia on phagocytosis, cytokine expression, and transcription factor activation in peripheral blood cells.

Short-term hypoxia, equivalent to a height of 5500 m in a normobaric hypoxia chamber, did increase EPO concentration significantly without affecting plasma HIF-1 or serum VEGF concentrations. We found a considerable variability of plasma HIF-1 induction by hypoxia ranging from 0.13 to 21.0 fold. The fact that we did not find HIF-1 activation in PBMC is in good agreement with data published by Jiang et al. [20]. This group determined a significant HIF-1 activation with oxygen concentrations below 10% in HeLa cells, an oxygen concentration value not achieved in our experiment. Our results also confirm data published by Mounier et al. in 2009 [21], who examined the concentration of HIF-1*α* mRNA in leukocytes, serum EPO concentration, and plasma VEGF concentration after hypoxia induced in a hypoxia chamber with an oxygen concentration equivalent to a height of 3000 m in endurance athletes. Under these conditions, HIF-1*α* mRNA was significantly increased in leukocytes and serum EPO concentrations were increased whereas VEGF decreased. Similar effects of hypoxia on leukocyte HIF-1 DNA binding and protein concentration were observed by Tissot van Patot et al. in a hypoxia chamber [22]. In the present study, we found a significant increase of HIF-1 protein in the PBMC cytosol, although no increase of HIF-1 protein in the nucleus was observed indicating that under hypoxia, despite an increase of cytosolic HIF-1, protein translocation in the nucleus is separately regulated. Because leukocytes were examined in the former studies and PBMC in our study, we speculate that the effect of hypoxia on blood cells may be similar in all cell types. 

Mounting evidence over the last few years points to an association between the HIF and NF*κ*B pathways. The inhibition of oxygen-dependent hydroxylases under hypoxic conditions results in several consequences. On the one hand, phosphorylation of I*κ*B mediates the activation of p50/p65, and, on the other hand, this prevents HIF-1*α* from undergoing a proteasomal degradation that would lead to increased concentrations of HIF-1*α*. Since a NF*κ*B binding site is present in the promoter for the HIF-1*α* gene, NF*κ*B can increase HIF-1*α* mRNA and protein [23, 24]. Indeed, we found a raised activation of the NF*κ*B pathway in hypoxic PBMC of healthy volunteers as well as an increased cytosolic HIF-1 concentration which may be explained by the crosstalk between NF*κ*B and HIF-1. Nevertheless, this crosstalk does not account for the observation that increased cytosolic HIF-1 levels were not followed by an increase in HIF-1 nuclear concentration. Thus, we speculate that translocation of HIF-1*α* into the nucleus is also regulated and was not apparent under our experimental conditions. A further regulatory step in HIF-1*α*-induced gene expression may also explain the fact that under hypoxia we found only TNF*α* decreased in monocytes and IFN in CD4^+^ T lymphocytes whereas all other tested cytokines were not affected by hypoxia.

As HIF-1 is able to regulate EPO and VEGF expression, we examined these concentrations under hypoxia. Comparable to Mounier et al. [21] and Eckardt et al. [25], we found a significant increase in serum EPO concentration after hypoxia. Whilst Mounier described a significant decrease of plasma VEGF concentration after 3 h, we noted only a modest and not a significant reduction in serum VEGF levels. This discrepancy might be attributed to the duration of hypoxia or even to the number of volunteers which was too small to reach significance and awaits further investigation. Finally, comparable to other investigators [25–27], our results revealed a marked individual variation of the serum EPO concentration under hypoxia (increase of 1.44-to 21.18-fold by hypoxia) indicating an individual response to hypoxia. 

Phagocytosis by macrophages and neutrophils is the organism's first line of defence against bacterial infection. Systemic inflammatory diseases are characterised by the development of hypoxia, a process in which clearance of bacteria is of particular importance. For example, hypoxia often develops subsequent to major burns [28], trauma [29], and pancreatitis [30], often followed by a bacterial superinfection. This clinical observation may suggest that hypoxia is critical in phagocytosis. Anand et al. showed in mouse macrophages [31] that under hypoxia, phagocytosis increases, is positively regulated by p38 phosphorylation and augments HIF-1*α* expression. In the present study, systemic hypoxia caused an increase in phagocytosis in neutrophils as measured by the uptake of FITC-labeled zymosan. Our results are in good agreement with Wang and Chiu [10] who clearly demonstrated that under hypoxic conditions phagocytosis in neutrophils as well as chemotaxis and ROS production is enhanced. Increased phagocytosis of neutrophils may be a counterregulatory process to clean hypoxic areas from invading organisms.

The explanation relating to *in vivo* monocyte and CD4^+^ T lymphocyte cytokine production under hypoxic conditions provides a slight difficulty. Oxygen concentrations equivalent to a height of 5500 m for 2 hours caused a decrease of both TNF*α* in monocytes and INF in CD4^+^ T lymphocytes in our subjects as analysed using FACS. We used a cytokine array which is able to distinguish between a Th1- and Th2-inflammatory response in CD4^+^ T lymphocytes. Under hypoxia, we found a nonsignificant shift to a Th2-inflammatory response in CD4^+^ T lymphocytes. The fact that after stimulation with PMA/iono this response is abolished showed that hypoxia did not alter cellular T-cell activation. Thus, our *in vivo* results are in contrast to former *in vitro* studies. Ghezzi et al. [32] found an increased secretion of TNF*α*, IL-1*α*, and IL-1*β* after severe hypoxia and stimulation with PMA in PBMC of healthy volunteers. It was concluded that hypoxia renders PBMC more susceptible to external stimuli. But these authors did not differentiate between monocytes and lymphocytes in their experiments. An aggravating effect of TNF*α* production by hypoxia was also reported by Demasi et al. [33], who found an increased TNF*α* synthesis in LPS-stimulated monocytes of healthy blood donors under hypoxic culture conditions. In T lymphocytes of different mouse strains, Roman et al. [18] described an increased IFN production after activation with antibodies against CD3 and CD28 in severe hypoxia. Interestingly, this increase in INF production was also observed in T lymphocytes of mouse strains lacking one copy of the gene encoding HIF-1*α* indicating that this increase is independent of HIF-1*α* activation. 

Taken together, most published studies show that hypoxia in in vitro sensitized monocytes and T-lymphocytes represents an activating stimulas resulting in increased production of inflammatory cytokines. Though discrepancies relating to cytokine production are apparent, these can be explained by the fact that most groups used PBMC, monocytes or CD4^+^ T lymphocytes of healthy donors which were incubated in cell cultures under hypoxic conditions. We, in contrast, used PBMC from healthy donors who were exposed to hypoxia in a hypoxia chamber for several hours. The PBMCs were subsequently incubated for 6 h under normoxic conditions without or with PMA/iono. The very different experimental settings may lead to the different cellular behaviour following the stimulation of PMBC. Finally, our results are in good agreement with a study published by Dziurla et al. [19]. Similar to our data, the authors found a significant decrease of TNF*α* in CD4^+^ T lymphocytes, although the effect of CD4^+^ T lymphocyte production of INF was not tested by this group. Under *in vivo* conditions, glucose, which has a negative effect on cytokine production [19], is not involved since we could show that under short-term hypoxia in a hypoxia chamber, glucose concentration was not affected.

Taken together, our study demonstrates that moderate short-term systemic hypoxia (1) increases phagocytosis in neutrophils and not in monocytes, (2) diminishes TNF*α* production in monocytes and INF production in CD4^+^ T lymphocytes, (3) increases HIF-1*α* protein expression in PBMC without translocation into the nucleus, and (4) activates the NF*κ*B pathway in these cells. In addition, the proposed effect of hypoxia in PBMC on HIF-1*α* and NF*κ*B pathway activation was demonstrated in the current study and supports the crosstalk between HIF and NF*κ*B signaling in short-term hypoxia 

**Figure 8:**
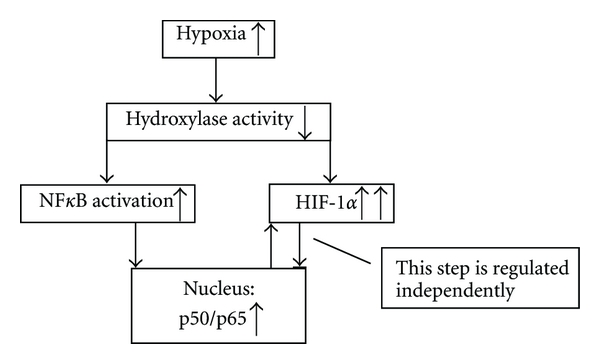
Proposed mechanism of hypoxia on HIF-1 and NF*κ*B activation in PBMC. Hypoxia causes a reduction of hydroxylase activity leading on the one hand to the activation of NF*κ*B and a consecutive transport of the p50/p65 complex into the nucleus. Binding of p50/p65 to the corresponding promotor side of the HIF-1*α* gene results in an increase of HIF-1*α* protein in the cytosol. On the other hand, reduced hydroxylase activity prevents HIF-1*α* degradation by the proteasome increasing cytosolic HIF-1*α* concentration. Under hypoxic conditions equivalent to a height of 5500 m, an increase of cytosolic HIF-1*α* did not raise the HIF-1*α* concentration in the nucleus indicating that not only the HIF-1 concentration in the cytosol, but also the transport into the nucleus is regulated.

Thus, we hypothesise that the effects of hypoxia under our experimental setting were due to NF*κ*B activation. Furthermore, our results suggest that in mild short-term hypoxia, HIF-1 translocation into the nucleus is separately regulated (Figure 8).

## Figures and Tables

**Table 1:** Subject characteristics at baseline and under hypoxia equivalent to a height of 5500 m for 2 hours.

	Normoxia	Hypoxia
Age (y)	30 ± 6	
Height (cm)	178 ± 8	
Weight (kg)	75 ± 13	
Heart rate (beats/min)	73 ± 9	87 ± 11*
Systolic blood pressure (mmHg)	129 ± 10	119 ± 16*
Diastolic blood pressure (mmHg)	82 ± 6	73 ± 10*
O_2_ saturation (%)	97 ± 2	78 ± 3*
Breathing frequency (per min)	13 ± 2	21 ± 6*

Values are mean ± SD, *n* = 14, *P < .05 normoxia versus hypoxia.

**Table 2:** Electrolytes, glucose, and lactate serum concentrations at baseline and under hypoxia equivalent to a height of 5500 m for 2 hours.

	K	Na	Ca	Cl	Glu	Lac
Normoxia	4.4 ± 0.7	145 ± 8	1.1 ± 0.1	109 ± 6	5.4 ± 0.7	1.8 ± 1.0
Hypoxia	4.2 ± 1.0	142 ± 6	1.1 ± 0.1	105 ± 5	5.1 ± 0.7	2.2 ± 0.6

Values are mean ± SD, *n* = 14.

## Abbreviations

EPO: Erythropoietin
HIF: Hypoxia inducible factor
IL: Interleukin
INF: Interferon *γ*
NF*κ*B: Nuclear factor kappa B
ROS: Reactive oxygen species
TNF*α*: Tumour necrosis factor *α*
VEGF: Vascular endothelial growth factor.
